# Hybrid Physical Examination: Integrating Peer Feedback with Standardized Patient Encounters

**DOI:** 10.1007/s40670-026-02751-2

**Published:** 2026-05-07

**Authors:** Shivani Srivastava, Sophia Chen, Rachel Rosenberg, Christin Traba, Jeremy Grachan, Robyn Kampf

**Affiliations:** Rutgers New Jersey Medical School, 185 South Orange Avenue, Newark, NJ 07103 USA

**Keywords:** Standardized patients, Peer feedback, Physical examination, Feedback literacy, Undergraduate medical education

## Abstract

**Context:**

Physical examination skills are fundamental to clinical practice, yet traditional training models often fail to integrate structured peer feedback with standardized patient (SP) encounters, limiting development of both clinical and feedback competencies.

**Objectives:**

This study evaluated a novel hybrid physical examination training model that integrates structured peer feedback with SP encounters across multiple organ systems, hypothesizing that this approach would improve students’ physical examination knowledge, confidence, and feedback literacy.

**Methods:**

This prospective educational intervention involved a cohort of 175 preclinical students at a U.S. medical school across four systems (musculoskeletal, cardiovascular, pulmonary, gastrointestinal) during 2025. Each session comprised facilitator-led peer practice using standardized checklists, followed by SP encounters where students rotated through examiner and observer roles, providing structured oral and written feedback. Pre- and post-session surveys assessed confidence and perceptions using a 5-point Likert scale, while ungraded knowledge tests measured skill acquisition. Data were analyzed using Mann-Whitney U tests.

**Results:**

Knowledge scores improved significantly in musculoskeletal (MSK) (4.02 to 4.28, *p* = 0.048), cardiovascular (CV) (3.77 to 4.18, *p* = 0.018), and gastrointestinal (GI) (3.47 to 4.05, *p* = 0.028) sessions. Confidence in performing physical examinations increased significantly across all systems (all *p* < 0.001). Perceptions of SP educational value improved in MSK (4.26 to 4.69, *p* < 0.001), CV (4.41 to 4.71, *p* = 0.009), and GI (4.29 to 4.95, *p* < 0.001) sessions. Ratings for both giving and receiving feedback improved significantly in most sessions. Pulmonary session results showed confidence gains but non-significant changes in other measures, likely due to reduced response rates.

**Conclusions:**

The hybrid model effectively enhanced physical examination knowledge, confidence, and feedback literacy across multiple organ systems. Integration of structured peer feedback with SP-based practice prepares students for collaborative and feedback-rich clinical environments. This model shows promise for strengthening skill acquisition and feedback competence in undergraduate medical education.

## Introduction

Physical examinations are a fundamental skill in clinical practice, yet modern medical curricula often struggle to provide consistent opportunities for students to develop and refine these skills through structured feedback [1, 2]. This gap represents a critical challenge in medical education: how to simultaneously develop physical examination proficiency and the feedback literacy essential for lifelong learning and professional development.

Traditional training models typically fall into three categories, each with distinct limitations. Independent practice offers flexibility but lacks formal feedback mechanisms. Small-group sessions with peer and facilitator input promote collaborative learning but may feel artificial when students practice with classmates in peer physical examinations (PPE). Standardized patient (SP) encounters replicate clinical practice but can underemphasize the development of students’ feedback and self-assessment skills [3, 4].

Although SP-based instruction has been demonstrated to improve student confidence and clinical communication, it often relies heavily on feedback from SPs alone [5, 6]. This feedback can vary in quality and depth depending on SP training and individual interpretation [7]. Furthermore, traditional SP encounters rarely harness the full potential of peer-to-peer feedback, which has been shown to promote professional identity formation, reflective practice, and critical thinking in medical learners [8–10]. This represents a missed opportunity, as peer feedback not only reinforces clinical skills but also develops the giving and receiving of feedback.

### Knowledge Gap and Study Rationale

Despite substantial evidence supporting both peer-assisted learning and SP-based education independently, no studies have systematically integrated structured peer feedback into SP-based physical examination training across multiple organ systems throughout the preclinical curriculum. This gap is significant because feedback literacy, defined as the capacity to critically evaluate one’s own performance and that of others, is foundational to self-regulated learning and professional development [11]. Furthermore, combining these modalities may create synergistic effects. The authenticity of SP encounters enhances engagement, while peer feedback deepens cognitive processing through observation, evaluation, and articulation of performance standards.

The foundation for this integration draws from deliberate practice and feedback-informed learning frameworks [12, 13]. Deliberate practice emphasizes repetition with immediate, specific feedback on performance [14]. Feedback-informed learning positions students as active agents in seeking, interpreting, and utilizing feedback [15]. By engaging students simultaneously as both performers and evaluators, this hybrid approach promotes metacognitive awareness and self-regulation. These skills transfer directly to clinical practice where physicians must continuously self-assess and learn from colleagues.

### Current Study

To address this gap, we developed and evaluated a longitudinal hybrid teaching model integrating structured peer feedback into SP-based physical examination sessions. Implemented across the 2025 academic year, the model spans four organ systems, including musculoskeletal (MSK), cardiovascular (CV), pulmonary, and gastrointestinal (GI). In each session, students first practice physical examination techniques on peers in facilitator-led sessions using standardized checklists aligned with curricular objectives. They then apply these skills in SP encounters, rotating through performance and observation roles to deliver and document structured oral and written feedback using the same checklists.

This dual-role approach during the SP encounters promotes authenticity through SP engagement while empowering learners to critically observe, evaluate, and articulate performance through peer feedback. By engaging students in both giving and receiving feedback, this model aims to enhance not only physical examination proficiency and confidence but also feedback skills.

This study evaluated the impact of the hybrid model on one cohort of medical students throughout their preclinical curriculum, focusing on improvements in physical examination knowledge, confidence, and feedback competence across multiple body systems. The hypothesis is that the integration of structured peer feedback into SP encounters will improve student learning outcomes compared to traditional approaches while fostering collaborative learning and self-regulated skill development. Findings will support ongoing efforts to redesign physical examination training in undergraduate medical education, with potential broader implications for enhancing clinical readiness and lifelong learning capabilities.

## Methods

### Study Design and Ethics

This prospective educational intervention study was approved by the institutional review board of a U.S. medical school and was deemed exempt due to minimal risk. Digital informed consent was obtained before each session. Participation was voluntary, explicitly separated from course evaluations and grading, and had no bearing on students’ academic standing or course performance. Students retained the right to decline or withdraw at any point without consequence.

### Participants and Setting

This study involved a cohort of approximately 175 students enrolled in a longitudinal preclinical doctoring course at a U.S. medical school in 2025. Eligibility was limited to students enrolled in the course who agreed to complete voluntary, ungraded surveys and knowledge tests.

### Intervention

The intervention consisted of four physical examination sessions across the Spring (year one preclinical) and Fall 2025 (year two preclinical) semesters, targeting the MSK, CV, pulmonary, and GI systems. The cohort completed the MSK, CV, and pulmonary sessions as first-year students in Spring 2025, followed by the GI session in Fall 2025 after transitioning to the second year. These four organ systems were delivered as four standalone sessions. Renal, genitourinary, neurology, psychiatric, and behavioral systems were excluded based on curricular timing constraints and the sensitivity of certain examinations that require specialized training and standardized assessment protocols.

**Fig. 1:**
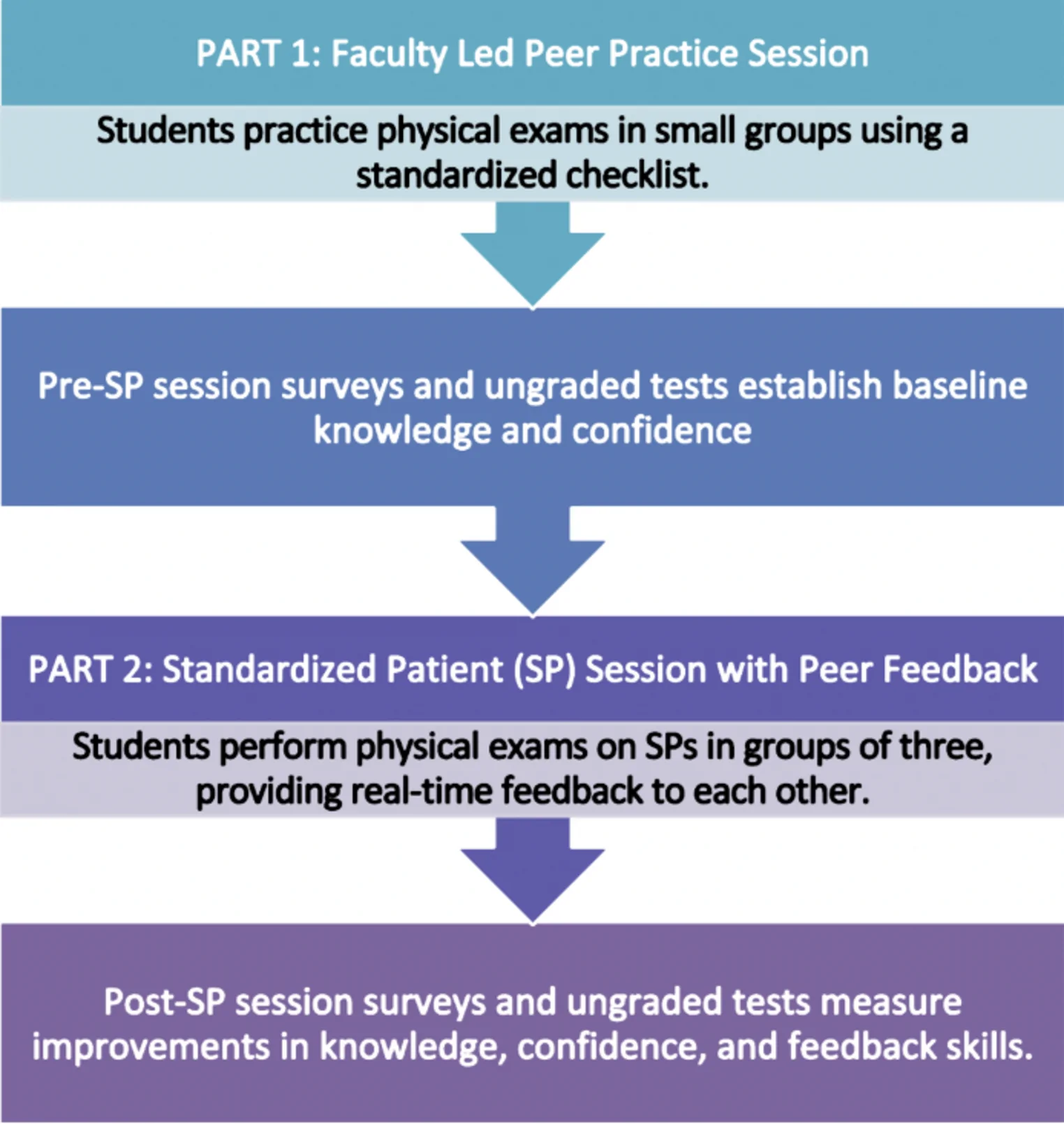
Workflow of the longitudinal hybrid teaching model. Students first complete faculty-led peer practice sessions using standardized checklists, followed by standardized patient (SP) encounters in which students rotate between examiner and observer roles to provide structured peer feedback. Pre- and post-SP session surveys and ungraded knowledge tests are used to assess confidence, knowledge, and feedback skills

Each physical examination session had a standardized two-part format (Fig. 1). Part 1 consisted of a two-hour facilitator-led peer practice session. Students participated in small groups (6–8 students) to practice system-specific physical examination skills on their peers. Clinical faculty guided students using standardized checklists aligned with curricular objectives. During this practice, students used the checklists while examining peers, learning both procedural skills and assessment criteria.

Part 2 consisted of one-hour standardized patient (SP) encounters with peer feedback, during which students participated in SP sessions conducted in groups of three. In each group, one student served as the examiner while the remaining two served as observers. Students rotated roles so that each student served as examiner once and observer twice per session. One SP was assigned to each group. Observers provided structured oral and written feedback using the same checklist used in Part 1, reinforcing consistent evaluation standards and feedback skills. Checklists embedded in the preclinical curriculum ensured uniformity in peer evaluations during SP encounters.

### Data Collection

Data were collected twice during each SP session using secure QR code-linked electronic surveys. Before the SP encounter, students completed a pre-session survey measuring confidence and perceptions using a 5-point Likert scale. This scale addressed confidence in performing system-specific examinations, perceived educational value of SP encounters, and attitudes toward providing and receiving peer feedback, along with a five-question ungraded knowledge test composed of lower-order multiple-choice questions.

After the SP encounter, students completed a post-session survey and the same five-question knowledge test to evaluate changes in knowledge and confidence. The post-session survey included all pre-session questions plus additional Likert items and open-ended questions to capture qualitative feedback on the session’s structure, content, and perceived educational value.

All survey responses were screened using anonymous identifiers to verify survey validity and remove incomplete or invalid entries. After this verification step, identifiers were removed and responses were de-identified. Pre-session and post-session responses were then analyzed as independent samples. Response counts are reported as n_Pre_ (number of students completing the pre-session survey) and n_Post_ (number of students completing the post-session survey). Because participation was voluntary and surveys were anonymous, pre- and post-session samples could not be individually matched.

### Data Analysis

Quantitative data were analyzed using IBM SPSS Statistics (Version 29). Normality was assessed using the Shapiro-Wilk test. Descriptive statistics summarized responses, and Mann-Whitney U tests were used for pre- and post-session comparisons due to non-normal distributions and because pre- and post-session responses were analyzed as independent samples. Qualitative data from open-ended responses were independently coded by two research team members using an inductive approach, then compared to establish consensus themes.

## Results

### Survey Response Rates

Response rates varied across the four sessions. For the MSK session, 129 of approximately 175 students completed the pre-session survey (73.7%) and 108 completed the post-session survey (61.7%). In the CV session, 71 students responded pre-session (40.6%) and 51 post-session (29.1%). In the pulmonary session, 26 students completed the pre-session survey (14.9%) and 11 completed the post-session survey (6.3%). The GI session had 34 pre-session respondents (19.4%) and 20 post-session respondents (11.4%). Across all sessions, post-session response rates were lower than pre-session rates.

### Knowledge of Physical Examination

Knowledge scores from the five-question ungraded knowledge test improved significantly in three of the four sessions (Table 1). Musculoskeletal (MSK) session mean scores rose from 4.02 to 4.28 (n_Pre_ = 129, n_Post_ = 108, *p* = 0.048). Cardiovascular (CV) session knowledge increased significantly from 3.77 to 4.18 (n_Pre_ = 71, n_Post_ = 51, *p* = 0.018). Gastrointestinal (GI) knowledge improved from 3.47 to 4.05 (n_Pre_ = 34, n_Post_ = 20, *p* = 0.028). In the pulmonary session, knowledge changes were not statistically significant (3.65 to 3.82, n_Pre_ = 26, n_Post_ = 11, *p* ≥ 0.29).

**Table 1:** Pre- and post-SP session confidence, standardized patient usefulness, peer feedback perceptions, and ungraded knowledge test scores across four physical examination systems

Measure	Pre *n*	Post *n*	Pre Mean ± SD	Post Mean ± SD	*P*-Value (Mann-Whitney U)
Musculoskeletal					
Upper Extremity Confidence	129	108	2.61 ± 1.03	4.33 ± 0.63	< 0.001***
Lower Extremity Confidence	129	108	2.53 ± 0.99	4.19 ± 0.73	< 0.001***
SP Usefulness	129	108	4.26 ± 0.82	4.69 ± 0.51	< 0.001***
Receiving Verbal Feedback	129	108	4.29 ± 0.67	4.63 ± 0.54	< 0.001***
Receiving Written Feedback	129	108	4.12 ± 0.71	4.45 ± 0.72	< 0.001***
Giving Verbal Feedback	129	108	4.26 ± 0.56	4.54 ± 0.59	< 0.001***
Giving Written Feedback	129	108	4.26 ± 0.56	4.45 ± 0.69	< 0.001***
Ungraded Test Score	129	108	4.02 ± 1.16	4.28 ± 1.08	0.048*
Cardiovascular					
CV Examination Confidence	71	51	2.92 ± 1.01	4.29 ± 0.70	< 0.001***
SP Usefulness	71	51	4.41 ± 0.69	4.71 ± 0.50	0.009**
Receiving Verbal Feedback	71	51	4.31 ± 0.72	4.59 ± 0.57	0.007**
Receiving Written Feedback	71	51	4.20 ± 0.77	4.49 ± 0.65	0.009**
Giving Verbal Feedback	71	51	4.13 ± 0.75	4.53 ± 0.61	0.003**
Giving Written Feedback	71	51	4.08 ± 0.82	4.49 ± 0.65	< 0.001***
Ungraded Test Score	71	51	3.77 ± 0.89	4.18 ± 0.92	0.018*
Pulmonary					
Pulmonary Examination Confidence	26	11	2.50 ± 0.76	4.45 ± 0.52	< 0.001***
SP Usefulness	26	11	4.10 ± 0.74	4.20 ± 0.63	≥ 0.29 NS
Receiving Verbal Feedback	26	11	4.20 ± 0.71	4.40 ± 0.70	≥ 0.29 NS
Receiving Written Feedback	26	11	4.30 ± 0.67	4.60 ± 0.52	≥ 0.29 NS
Giving Verbal Feedback	26	11	4.10 ± 0.74	4.20 ± 0.63	≥ 0.29 NS
Giving Written Feedback	26	11	4.10 ± 0.74	4.20 ± 0.63	≥ 0.29 NS
Ungraded Test Score	26	11	3.65 ± 0.89	3.82 ± 0.98	≥ 0.29 NS
Gastrointestinal					
GI Examination Confidence	34	20	2.71 ± 0.91	4.55 ± 0.51	< 0.001***
SP Usefulness	34	20	4.29 ± 0.69	4.95 ± 0.22	< 0.001***
Receiving Verbal Feedback	34	20	4.15 ± 0.74	4.75 ± 0.44	< 0.001***
Receiving Written Feedback	34	20	4.03 ± 0.83	4.45 ± 0.69	< 0.001***
Giving Verbal Feedback	34	20	4.12 ± 0.78	4.80 ± 0.41	< 0.001***
Giving Written Feedback	34	20	4.06 ± 0.85	4.50 ± 0.61	< 0.001***
Ungraded Test Score	34	20	3.47 ± 0.96	4.05 ± 1.05	0.028*

*p < 0.05; **p < 0.01; ***p < 0.001; NS = not significant. Mann-Whitney U tests were conducted on independent pre-session and post-session samples. Confidence and perception measures used 5-point Likert scales (1 = strongly disagree, 5 = strongly agree). Ungraded test scores ranged from 0 to 5

### Confidence in Physical Examination Performance

**Fig. 2:**
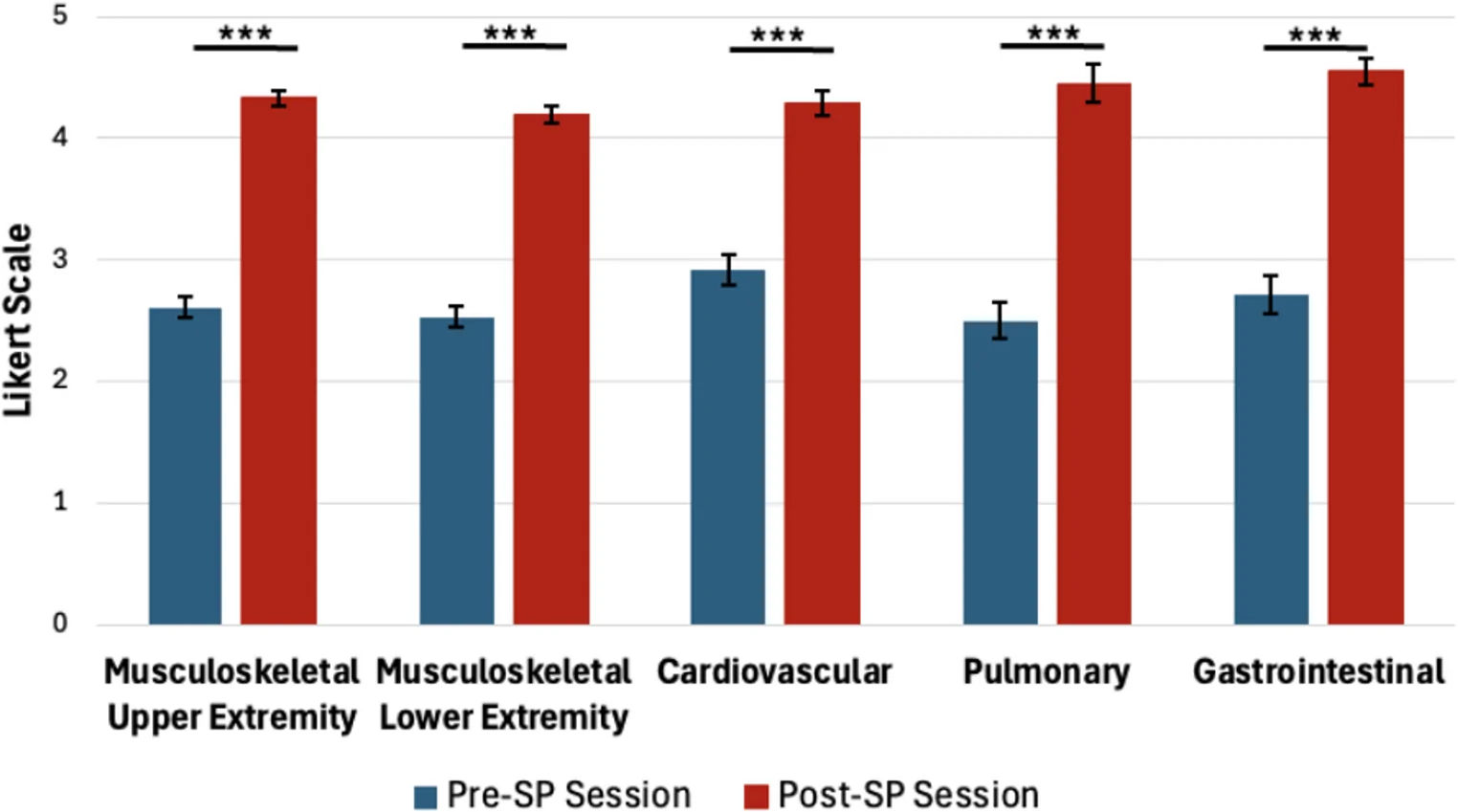
Pre- and post-SP session confidence ratings across five physical examination categories (musculoskeletal upper extremity, musculoskeletal lower extremity, cardiovascular, pulmonary, gastrointestinal). Confidence was measured using a 5-point Likert scale (1 = strongly disagree to 5 = strongly agree). Error bars represent standard error of the mean. Asterisks denote significance levels: *p < 0.05, **p < 0.01, and ***p < 0.001; *ns* = not significant

Student-reported confidence in performing physical examination maneuvers increased significantly across all systems (Fig. 2; Table 1). In MSK exams, confidence for the upper extremity rose from 2.61 to 4.33, and for the lower extremity from 2.53 to 4.19 (n_Pre_ = 129, n_Post_ = 108, both *p* < 0.001). CV examination confidence improved from 2.92 to 4.29 (n_Pre_ = 71, n_Post_ = 51, *p* < 0.001). Pulmonary examination confidence saw an increase from 2.50 to 4.45 (n_Pre_ = 26, n_Post_ = 11, *p* < 0.001). GI confidence also rose significantly from 2.71 to 4.55 (n_Pre_ = 34, n_Post_ = 20, *p* < 0.001).

### Perceptions of Standardized Patients

**Fig. 3:**
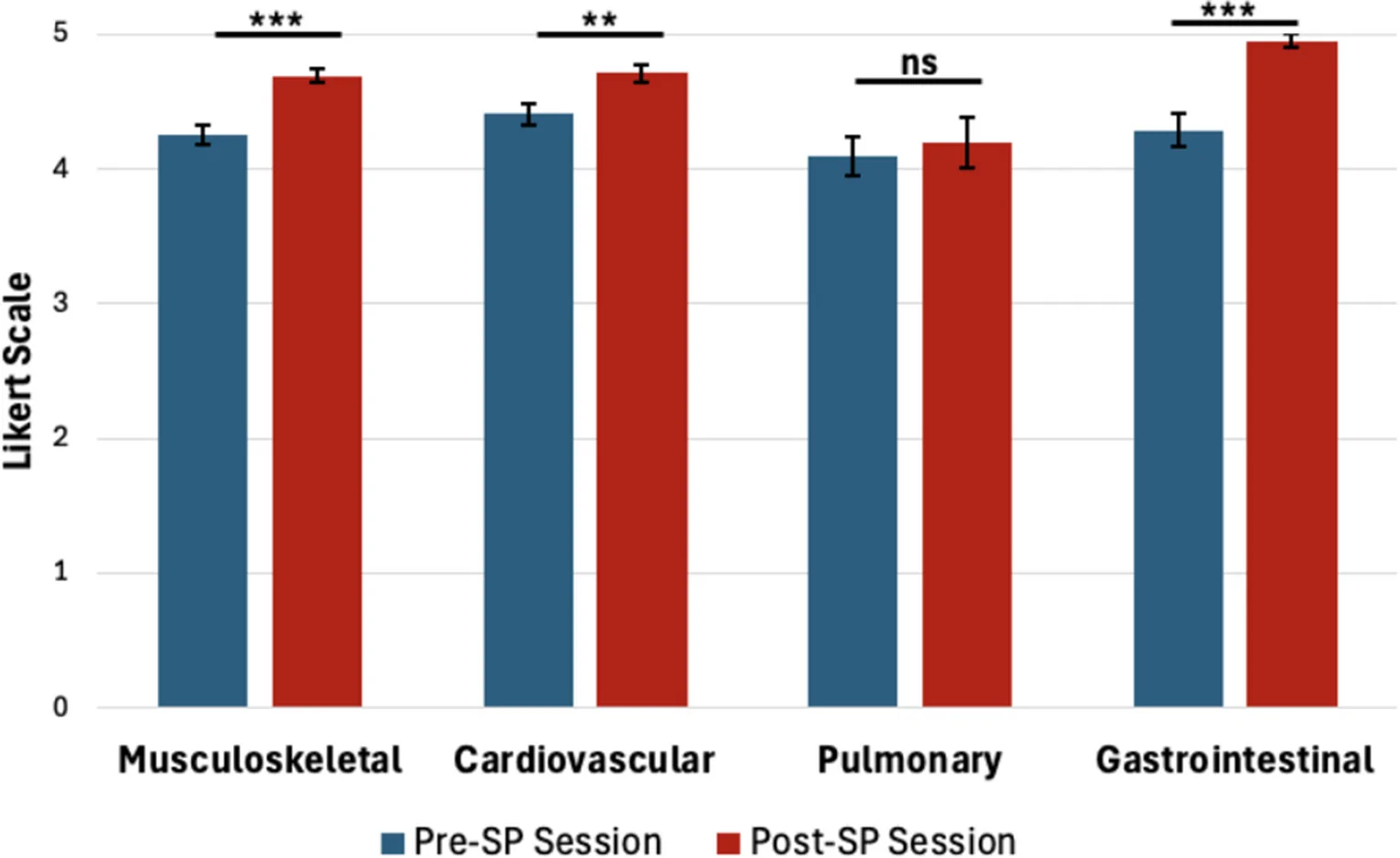
Pre- and post-SP session perceptions of standardized patient educational value across physical examination sessions. Ratings were measured using a 5-point Likert scale (1 = strongly disagree to 5 = strongly agree). Error bars represent standard error of the mean. Asterisks denote significance levels: *p < 0.05, **p < 0.01, and ***p < 0.001; *ns* = not significant

Students’ perceptions of the educational value of SP encounters improved significantly in three sessions (Fig. 3; Table 1). After the MSK session, ratings of SP usefulness increased from 4.26 to 4.69 (n_Pre_ = 129, n_Post_ = 108, *p* < 0.001). In the CV session, the perceived SP value rose from 4.41 to 4.71 (n_Pre_ = 71, n_Post_ = 51, *p* = 0.009). GI session also saw an increase in SP usefulness from 4.29 to 4.95 (n_Pre_ = 34, n_Post_ = 20, *p* < 0.001). In pulmonary sessions, students’ perceptions of SP sessions remained stable (n_Pre_ = 26, n_Post_ = 11, *p* ≥ 0.29).

### Perceived Benefits of Peer Feedback

**Fig. 4:**
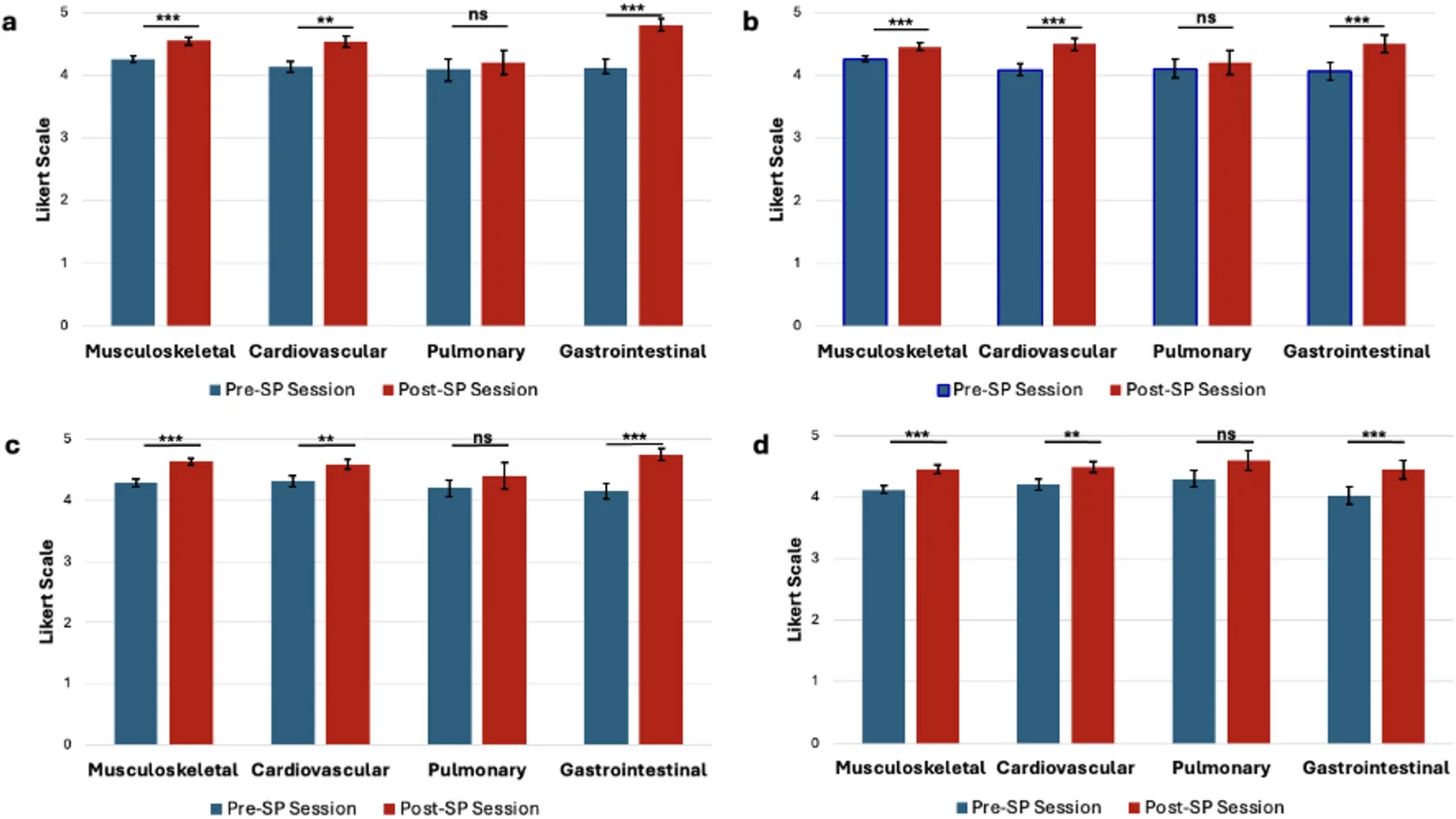
Pre- and post-SP session perceptions of peer feedback processes across four physical examination systems (musculoskeletal, cardiovascular, pulmonary, gastrointestinal). Ratings were measured using a 5-point Likert scale (1 = strongly disagree to 5 = strongly agree). Panels represent: (**a**) providing verbal peer feedback, (**b**) providing written peer feedback, (**c**) receiving verbal peer feedback, and (**d**) receiving written peer feedback. Error bars represent standard error of the mean. Asterisks denote significance levels: *p < 0.05, **p < 0.01, and ***p < 0.001; *ns* = not significant

Ratings on the perceived benefits of receiving and providing peer feedback (via standardized checklists) improved significantly in most areas (Fig. 4; Table 1). For MSK sessions, students reported increased benefit from receiving verbal (4.29 to 4.63, n_Pre_ = 129, n_Post_ = 108, *p* < 0.001) and written feedback (4.12 to 4.45, *p* < 0.001). Similarly, they reported increased benefit from giving verbal feedback (4.26 to 4.54, *p* < 0.001) and written feedback (4.26 to 4.45, *p* < 0.001). CV sessions showed similar improvements for both receiving and giving feedback across modalities (n_Pre_ = 71, n_Post_ = 51, all *p* < 0.01). GI exams produced large gains in both categories (n_Pre_ = 34, n_Post_ = 20, all *p* < 0.001). In pulmonary sessions, changes in feedback ratings, both giving and receiving, remained non-significant, though composite scores indicated overall positive perceptions (n_Pre_ = 26, n_Post_ = 11).

### Student Preparation

**Fig. 5:**
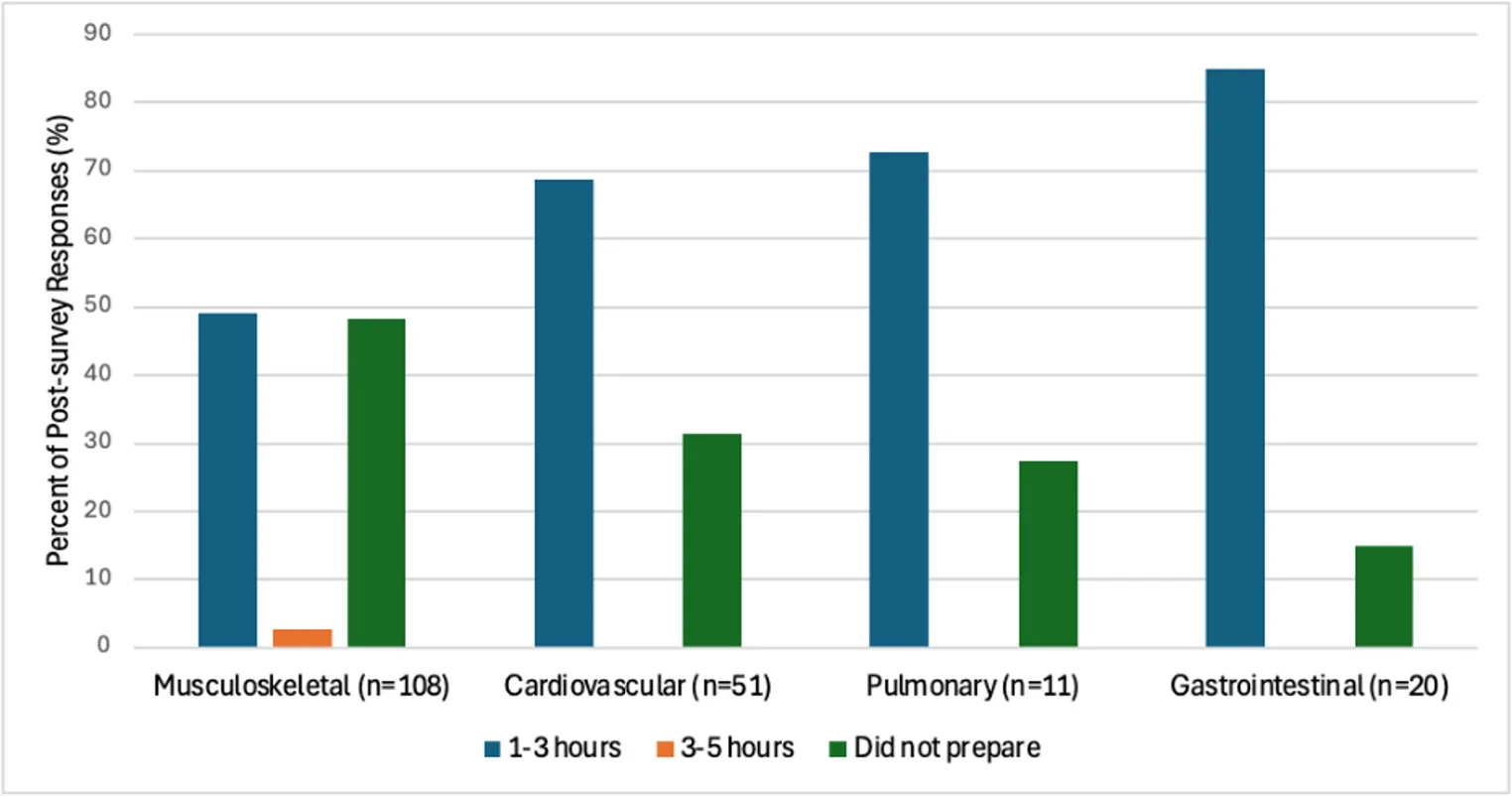
Preparation time reported in post-SP session surveys across musculoskeletal, cardiovascular, pulmonary, and gastrointestinal SP sessions. Bars represent the percentages of students who spent 1–3 hours, 3–5 hours, or did not prepare prior to the session. Percentages reflect the proportion of respondents completing the post-session survey for each system

Most students reported spending one to three hours preparing for each physical examination session (Fig. 5). Preparation rates were highest for the gastrointestinal (85%) and pulmonary (72.7%) sessions, followed by the cardiovascular (68.6%) and musculoskeletal (49.1%) sessions.

### Session Feedback

Responses to the question “How can the SP session with peer feedback be improved?” were analyzed using thematic analysis. This question was optional for students to answer, and responses from all four systems were aggregated. Of all post-session surveys collected across the four sessions (*n* = 190), 74 students chose to respond to the optional open-ended improvement question. Two research team members independently coded qualitative data using an inductive approach and met to reconcile their findings, resulting in six final themes (Table 2).

**Table 2:** Thematic analysis of responses to “How can the SP session with peer feedback be improved?” (*n* = 74)

Theme	Example Comments	*n* (%)
Positive perceptions of the session	Students described the sessions as “very well organized,” “good as is,” and “really helpful for learning physical examination skills.”	16 (21.6)
Time management	Suggestions to adjust session length or pacing, such as “15 min was a bit too much time for the exam” and “longer time for feedback.”	16 (21.6)
Checklist adjustments	Requests for clearer, more objective checklists with space for comments: “add a section for special maneuvers” and “include a comments box.”	14 (18.9)
Have faculty present during SP session	Desire for expert guidance and demonstration: “have a facilitator or attending to verify maneuvers” and “someone who knows what they’re doing.”	11 (14.9)
SP provide feedback	Students valued direct input from standardized patients: “it would be helpful if SPs could provide some feedback.”	7 (9.5)
Other	Miscellaneous ideas, including improved instructional videos, more preparation time, or quieter spaces for practice.	10 (13.5)
Total		74 (100)

The most frequent themes were positive perceptions of the session (*n* = 16) and session scheduling (*n* = 16). Many students described the session as well organized, valuable, and confidence-building, while others suggested minor adjustments to the session length or scheduling to allow for smoother pacing and more efficient feedback exchange. Comments emphasizing the importance of having faculty present during SP sessions (*n* = 11) reflected a desire for expert demonstration, clarification of proper technique, and enhanced supervision. The theme of checklist adjustments (*n* = 14) included suggestions to refine or simplify the feedback forms, add comment sections, and incorporate more objective rating criteria. Some students also expressed interest in having standardized patients provide feedback (*n* = 7) to complement peer and faculty perspectives. Finally, the other category (*n* = 10) captured additional ideas, such as improving preparatory materials and creating quieter or more structured environments for practice.

## Discussion

This study evaluated a hybrid model integrating standardized patient (SP) encounters with structured peer feedback across four organ systems in preclinical medical education. The findings demonstrated significant improvements in physical exam knowledge, confidence, and feedback competence, supporting the effectiveness of combining peer-assisted learning with authentic SP-based practice.

### Key Findings and Interpretation

Data showed significant confidence improvements across all four systems. Differences between pre-session and post-session mean confidence ratings ranged from 1.55 to 1.95 points on a 5-point Likert scale (all *p* < 0.001). These gains align with prior literature demonstrating that SP-based education enhances student self-efficacy and clinical competence [16, 17]. Students report higher confidence performing skills with SPs versus peer practice alone, as SPs provide authentic clinical environments without risk to real patients [16]. This study extends prior work by demonstrating that integrating peer feedback with SP encounters produces confidence gains comparable to SP encounters alone.

Furthermore, knowledge test scores improved significantly in MSK, CV, and GI sessions, providing objective evidence complementing subjective confidence measures (Table 1). Students’ enhanced perceptions of SP educational value improved significantly in MSK, CV and GI sessions, suggesting that structured peer feedback enriched the SP experience, consistent with literature on multimodal feedback [17].

Significant improvements in perceptions of both giving and receiving peer feedback were observed in the MSK, CV and GI sessions. These improvements indicate growth in feedback literacy, defined as the capacity to effectively seek, understand, engage with, and use feedback for improvement [15, 18]. Findings support prior reports that structured peer feedback promotes both clinical skills and metacognitive awareness [19–21]. Standardized checklists used in this study likely facilitated this development by providing concrete observation criteria, addressing documented gaps in feedback training among medical students [22, 23].

### Conditions for Effective Peer Feedback

Several intervention features appeared to contribute to the effectiveness of peer feedback. Structured checklists provide specific, actionable criteria. This aligns with systematic reviews demonstrating that structured frameworks enhance feedback quality to levels comparable with faculty feedback [24–26].

The sequential design, with facilitator-led peer practice followed immediately by SP encounters, supported learning, as evidenced by significant knowledge and confidence gains, consistent with the principles of self-regulated learning [27, 28]. Clear preparatory work through facilitator-led practice ensures shared understanding of learning objectives and assessment criteria. This was a critical element that students often lack training in providing effective feedback [22, 29].

The psychological safety created by peer-level feedback (rather than hierarchical evaluation) appeared conducive to learning, as peers often provide more comfortable environments for discussing uncertainties and receiving constructive criticism [30]. Research indicates that peer feedback encourages improvements in professionalism, teamwork, and communication skills precisely because peers serve as credible sources for assessing behaviors that faculty may struggle to observe in large cohorts [31].

### Pulmonary Session Anomaly

The pulmonary session showed a contrasting pattern: significant confidence improvement but non-significant changes in SP and peer feedback perceptions. Multiple factors may have led to this. Reduced response rates (n_Pre_ = 26, n_Post_ = 11) lowered statistical power to detect subtler attitudinal shifts. Competing curricular demands, diminished novelty, survey fatigue, and logistical issues likely contributed to decreased student engagement. Such attrition is common in longitudinal educational interventions.

The pulmonary session may also represent a natural plateau in learning gains. By this third session, foundational examination and feedback skills were established during earlier MSK and CV sessions, reducing measurable perceptual shifts. Students may have already internalized key elements of the peer feedback process, making further gains less detectable. Additionally, the smaller pulmonary sample raises concerns about non-response bias, potentially limiting representativeness.

Interestingly, while one might expect similar plateau effects in the GI session, the intervening summer break before GI likely served as a refresher period, contributing to the significant gains observed. This temporal spacing may have enhanced engagement, a finding worth exploring in future research on optimal spacing of skills training in undergraduate medical education.

### Strengths and Limitations

This study’s key strengths include integration of standardized checklists across both peer practice and SP encounters, ensuring consistency in assessment criteria and feedback quality. Inclusion of multiple organ systems enhances comprehensiveness and demonstrates reproducibility of intervention across different content areas. The longitudinal design spanning most of a preclinical curriculum provides insight into sustained effects. Triangulation of quantitative and qualitative data strengthens the validity of findings. Finally, the study addresses a significant gap in the literature by systematically integrating structured peer feedback into SP-based physical examination training.

This study has several important limitations. Data from a single institution and cohort limit external validity and generalizability to other educational contexts. Response rates declined between pre- and post-session surveys across all four modules, not only in the pulmonary session. One possible explanation is that lower-performing or less satisfied students were less likely to complete post-surveys, which could mean the improvements in confidence and knowledge scores reported in this study are higher than what the full cohort would show. Because surveys were anonymous, it was not possible to compare respondents and non-respondents by performance level. Future implementations should consider strategies to improve completion rates, such as embedding surveys into session workflows, using course platforms to facilitate completion, or offering small incentives, while keeping participation voluntary.

Additionally, in the current study, role order data were not systematically recorded. Therefore, no post hoc analysis was conducted to examine whether performance outcomes differed based on the sequence in which students served as examiner or observer. This is a meaningful limitation, as students in groups of three may have observed one or two encounters before performing their own examination, introducing a potential order effect whereby later examiners may benefit from additional observation time [32]. If results were partly driven by this ordering rather than the intervention itself, the observed improvements may overestimate the true effect of peer feedback and SP sessions. Future studies should track role assignment order and conduct post hoc analyses to isolate its impact on performance outcomes.

Furthermore, reliance on self-reported measures introduces potential for over- or underestimation of actual gains in confidence. Aside from the ungraded five-question knowledge tests, the study lacks objective performance assessment, such as OSCE scores, to validate self-reported improvements. Additionally, variability in faculty training may have influenced instruction consistency across sessions. Students also did not receive formal preparation in providing peer feedback, which could contribute to inconsistency in feedback quality. Finally, the absence of a control group limits causal inference about the intervention’s effectiveness.

### Implications and Future Directions

Results demonstrated that combining peer feedback with SP encounters can enhance clinical examination confidence and feedback literacy. Significant improvements across knowledge (3 of 4 sessions), confidence (all 4 sessions), SP educational value perception (3 of 4 sessions), and feedback perception measures (3 of 4 sessions) are consistent with literature showing that peer-assisted learning with structured practice improves both clinical skills and self-confidence [33, 34]. This approach directly addresses calls in medical education literature for interventions that develop feedback literacy alongside clinical competence to prepare students for the collaborative, feedback-rich environment of clinical practice [18, 35].

Future research should address current limitations. Incorporating objective performance measures (e.g., OSCE scores, faculty evaluations) would provide stronger validation of self-reported improvements [36, 37]. Longitudinal tracking through clinical rotations could determine whether observed improvements persist and transfer to patient care settings [40]. Multi-institutional studies would enhance generalizability, and controlled trials comparing this hybrid model to traditional instructional approaches could strengthen causal inference.

Additional directions emerge from qualitative findings: 14.9% of qualitative respondents (*n* = 11 of 74) requested greater faculty presence during SP sessions, suggesting a need to explore optimal faculty involvement levels. The temporal spacing effect observed between pulmonary and GI sessions warrants investigation to identify ideal practice intervals. Providing formal feedback training before participation may further enhance quality and consistency [22, 38, 39]. Preparation rates also varied across modules, from 49.1% in MSK to 85% in GI, but the relationship between preparation time and performance or confidence gains was not formally analyzed. Future research should explore whether greater preparation time predicts larger improvements, which could inform study recommendations ahead of SP sessions.

Overall, this hybrid model shows promise for strengthening physical examination skill acquisition, feedback competence, and collaborative learning in undergraduate medical training. As healthcare increasingly emphasizes team-based care and continuous quality improvement, developing the capacity to give and receive feedback becomes essential for professional practice [40]. This hybrid approach offers a method for achieving these dual objectives while maintaining the authenticity and engagement of traditional SP-based learning.

## Conclusions

The hybrid model integrating structured peer feedback with standardized patient encounters enhanced physical examination knowledge, confidence, and feedback literacy across multiple organ systems. This approach prepares students for collaborative and feedback-rich clinical environments and shows promise for strengthening skill acquisition and feedback competence in undergraduate medical education.

## Data Availability

De-identified data supporting the findings of this study are available from the corresponding author upon reasonable request, subject to institutional review board approval.
